# The patient-related factors in revision procedures on tibia of patients with osteogenesis imperfecta treated with the Peter-Williams nail

**DOI:** 10.1186/s13018-023-03952-w

**Published:** 2023-07-26

**Authors:** Wenbiao Zhu, Yang Xiong, Bo Li, Hongjiang Yang, Cong Xing, Xiuzhi Ren, Guangzhi Ning

**Affiliations:** 1 International Science and Technology Cooperation Base of Spinal Cord Injury, Tianjin Key Laboratory of Spine and Spinal Cord Injury, Department of Orthopedics, Tianjin Medical University General Hospital, 300052 Tianjin, China; 2 Tianjin Key Laboratory of Spine and Spinal Cord Injury, 300052 Tianjin, China; 3 Department of Pediatric Orthopedics, Wuqing People Hospital, 301700 Tianjin, China

**Keywords:** Osteogenesis imperfecta, Tibia, Revision rate, Age of initial surgery, Classification, Deformity

## Abstract

**Objective:**

To investigate the patient-related factors that affect the revision rate for the tibia in patients with osteogenesis imperfecta treated with the Peter-Williams nail, and to explore the relationship between the risk factors and complications postsurgery.

**Methods:**

We retrospectively analysed the data of 211 patients (93 females (44.08%) and 118 males (55.92%)) with osteogenesis imperfecta treated with Peter-Williams. The factors affecting surgical revision were analysed by performing binary logistic regression. Then, a total of 211 patients with type III, type I or type IV OI were divided into five groups according to the results of regression. Statistical comparison of these groups was performed to further investigate the relationship between patient-related factors and revision procedures. Statistical comparison was also performed to analyse the relationship between the classification and postoperative complications.

**Results:**

Among the 211 patients who underwent surgery, 40 had type I OI, 109 had type IV OI, and 62 had type III OI. Binary logistic regression revealed that the classification (OR = 3.32, 95% CI 1.06–10.39, *P* = 0.039) and initial operation age (OR = 0.83, 95% CI 0.76–0.92, *P* < 0.001) were significantly correlated with revision procedures. In type III patients, the initial operation age was significantly correlated with revision procedures (*P* < 0.001), and the revision rate was lower in patients aged 9 to12 years (*P* = 0.001). In type I and IV patients, the initial operation age was not significantly correlated with revision procedures (*P* = 0.281). Classification had a significant effect on postoperative deformity (*P* = 0.003).

**Conclusions:**

The study reported that the age of initial surgery and classification were the influencing factors affecting the revision procedures of tibia in patients with osteogenesis imperfecta treated with the Peter-Williams nail. In patients with type III disease, the revision rate was lower individuals aged 9–12 years old, and a higher incidence of postoperative deformity was observed.

**Supplementary Information:**

The online version contains supplementary material available at 10.1186/s13018-023-03952-w.

## Background

Osteogenesis imperfecta (OI) is a group of genetic diseases characterized by bone fragility. Even among patients with the mildest cases of OI, the risk of long bone fracture is 100 times higher than that among healthy individuals [1]. Traditionally, OI has been classified as mild (type I), moderate (type IV), severe (type III), and fatal (type II) based on the severity of the disease [2, 3]. To date, a variety of clinical features have been reported to be associated with OI: increased bone fragility, osteoporosis, multiple fractures, blue sclera, progressive deafness, and joint laxity, among others [4]. Patients with type I have the mildest form of OI, and they hardly ever show symptoms throughout their lives. However, compression fracture of the vertebral body has been widely observed in patients with type I, leading to mild scoliosis in adults [5]. Type II is the most serious type of OI, thus, type II is not discussed in this article because almost all patients with type II die during the perinatal period. Type III is the most severe type of OI among surviving patients. Type IV, which is characterized by local sclerosis of bone, is a compilation of various forms of disease that are milder than type III.

Although the causes of OI are being revealed, there are not yet any fundamental treatments [6]. The main treatment methods for patients with types I and IV are comprehensive approaches such as surgical correction, drugs, and rehabilitation based on the classification and clinical conditions; these approaches, aim to provide the best long-term function and autonomy [7]. Although medical management involving the use of bisphosphonates has improved the outlook for patients with osteogenesis imperfecta, surgery provides the best option for preventing fractures and deformity [8]. In the past ten years, the field of femoral surgery in OI has been explored steadily, but research on tibial surgery is still very poor [9]. Despite a high rate of complications, intramedullary rodding has been proven to be the most successful treatment for the prevention and correction of fractures and deformities of long bones [10, 11]. Based on the use of different fixation rods, tibial implants can be divided into Peter-Williams nails, rush rods, modified rush rods, plate, screws, and Kirschner wires. Regarding the complication rate and cost, tibial surgery for Peter-Williams nail implantation is widely performed at our centre. The results of studies on individual-based surgery programs for patients with different types of OI have been inconclusive; the recommended age of surgery, the rate of complications, and the revision rate of patients who undergo tibial surgery remain uncertain. The purpose of this study is to determine which patient-related factors can affect the revision procedures of tibia in patients with osteogenesis imperfecta treated with the Peter-Williams nail and to investigate the relationship between the risk factors and complications.

## Materials and methods

### Study design and population

**Fig. 1:**
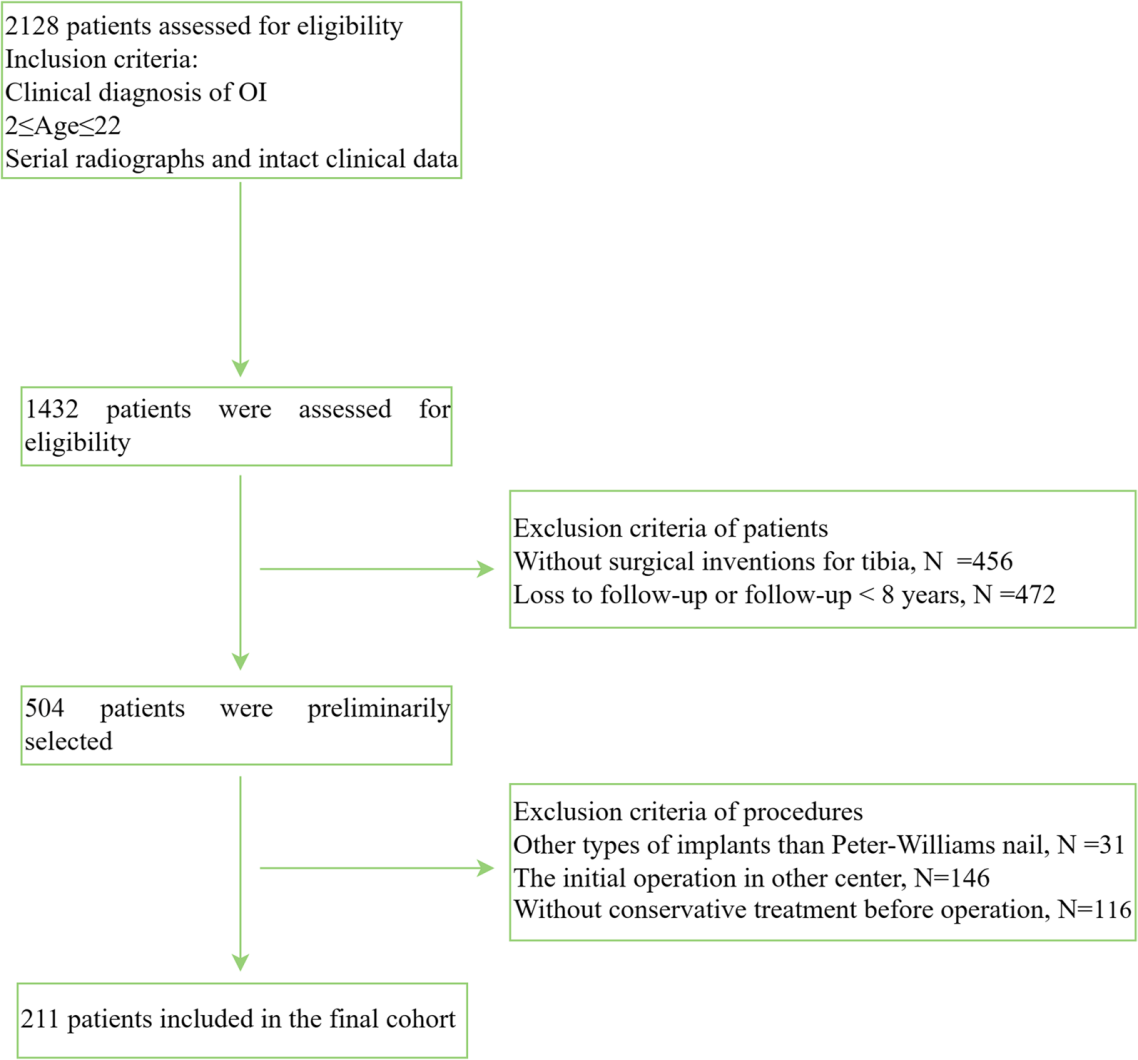
Flow chart of the inclusion and exclusion of this cohort study

This study was retrospective analysis of data from patients with OI managed at our centre. This article explored the effects of sex, the initial operation age, angle of the deformity and classification of OI on revision rate of patients treated with the Peter-Williams nail in the tibia diagnosed with lower extremity deformity. A total of 2128 patients with OI were followed clinically and radiologically at our centre from 2001 to 2021, and 211 patients with a diagnosis of OI were included based on the inclusion and exclusion criteria, as shown in (Fig. 1). All patients in our cohort had a history of conservative treatment with bisphosphonates and other drugs, and underwent initial operation with the Peter-Williams nail after conservative treatment failed, which was defined as the failure to achieve the ability to live independently despite restricted mobility. All procedures were conducted or followed up by our surgical team of three surgeons to avoid bias. All patients in the final cohort underwent conservative treatment with calcium and bisphosphonate before the initial operation with the Peter-Williams nail. The follow-up period was set at a minimum of 96 months and a maximum of 120 months. This article focused on the revision rate of ten years. In the final cohort, the retrospective study consisted of 211 patients (93 females (44.08%) and 118 males (55.92%)). Overall, 40 patients had Sillence type I, 62 patients had Sillence type III, and 109 patients had Sillence type IV. The following data were collected: sex, initial operation age, angle of the deformity, classification of OI, revision procedures, and incidence of complications. There were several complications associated with this procedure: refracture; deformity; and implant-related complications, including migration, breaking or bending of the implant, and nonunion of the bones. Most of the implant-related complications in this study were secondary to refracture or deformity, so refracture and deformity were recorded, and the location was distinguished. Then a total of 211 patients with type III, type I or type IV OI were divided into the following five age groups: less than or equal to 4 years old, from 5 to 8 years old, from 9 to 12 years old, from 13 to 16 years old and greater than or equal to 17 years old.

### Clinical classification

The classification was completed by three surgeons, and no significant difference was found. According to the revised Silence type of OI, 62 patients were type III (19 patients underwent revision, 43 patients did not undergo revision), and a total of 149 patients were type I and type IV (31 patients underwent revision and 118 patients did not undergo revision).

### Indication of surgical intervention

Patients with OI and tibial fractures or deformities older than 2 years were treated surgically, and patients younger than 2 years were typically treated conservatively. The following criteria had to be met for realignment osteotomies and Peter-Williams nail fixation in our centre: a tibial fracture or deformity; older than 2–3 years of age; more than 10 kg; the body is able to tolerate the operation; without other disease; and without operational contraindications. Due to unpredictable fractures and progressive deformities, revision procedures were necessary to replace or remove implants.

### Imaging data

Postoperative and follow-up radiographs including X-rays of the full-length (front and side) and CT of both lower limbs were evaluated to confirm osteotomy union. Standard radiographs were taken every 2 weeks until bone union was complete or the patients were revised and then once per month until the last review. According to the follow-up of the patients’ medical history and imaging data, the classification of participants was identified. The angle of the tibial deformity was measured using RadiAntviewer. Two researchers performed the measurements independently. Finally, the average value of the two measurements was taken and accurate to one decimal place.

### Statistical methods

Statistical analyses were performed using SPSS 25.0 (SPSS Inc., Chicago, Illinois, USA). The influencing factors were obtained through binary logistic regression. Normally distributed measurement data were expressed as the mean ± standard deviation, and non-normally distributed data was expressed as the median (P25, P75). The comparison of continuous variables (Initial operation age, Angle of deformity) between groups was performed by the Mann–Whitney U test. The comparison of categorical variables (Gender, Sillence classification) between groups was performed by the chi-square test. Statistical significance was set at *P* ≤ 0.05.

## Results

A total of 211 patients were included in the retrospective analysis, including 118 males and 93 females who underwent the Peter-Williams nail procedure (Table 1). Typical cases before and after the operation were shown in (Additional file 1: Fig. S1). To study the specific factors affecting postoperative revision, we included sex, initial operation age, angle, and classification in a binary logistic regression equation. The effect of initial operation age on revision was statistically significant (OR = 0.83, 95% CI 0.76–0.92, *P* < 0.001) (Table 2); the impact of type III on revision was statistically significant (OR = 3.32, 95% CI 1.06–10.39, *P* = 0.039) (Table 2) compared with type I, while the impact of type IV on revision was not statistically significant compared with type I. Sex had no significant effect on revision (OR = 0.63, 95% CI 0.32–1.27, *P* = 0.201) (Table 2), and the effect of angle on revision was not statistically significant (OR = 1.00, 95% CI 0.99–1.02, *P* = 0.700) (Table 2); The angle of preoperative deformity of the tibia did not have a significant effect on the revision rate.

**Table 1:** People demographics

Characteristic	Total (n = 211)	Revision (n = 50)	Non revision (n = 161)	p-value
Sex, n (%)				0.052^#^
Male	118(55.92)	22(18.64)	96(81.36)	
Female	93(44.08)	28(30.11)	65(69.89)	
Initial operation age (yrs)				< 0.001^^^
Median (IQR)	9.00(6.00)	10.00(6.00)	9.00(6.00)	
Mean (SD)	10.06(5.48)	11.19(5.52)	9.58(5.42)	
Angle				0.931^^^
Median (IQR)	29.80(21.10)	44.20(43.80)	27.50(15.25)	
Sillence, n (%)				
Type I	40(18.96)	6(12.00)	34(21.12)	0.068^#^
Type III	62(29.38)	19(38.00)	43(26.71)	
Type IV	109(51.66)	25(50.00)	84(52.17)	

SD = Standard Deviation, IQR: interquartile range. #Pearson's chi-square; ^Mann–Whitney U test

**Table 2:** Binary logistic regression equation

Characteristic	Group	B value	Standard error of B	Wald value	P value	OR value 95%CI of OR
Sex	Male	− 0.47	0.36	1.64	0.201	0.63 0.32–1.27
	Female					
Initial operation age		− 0.19	0.05	14.13	< 0.001	0.83 0.76–0.92
Angle		0.003	0.01	0.15	0.700	1.00 0.99–1.02
Sillence type III		1.20	0.58	4.26	0.039	3.32 1.06–10.39
Sillence type IV		0.54	0.53	1.05	0.305	1.72 0.61–4.84

OR = Odds ratio, CI = confidence interval

**Fig. 2:**
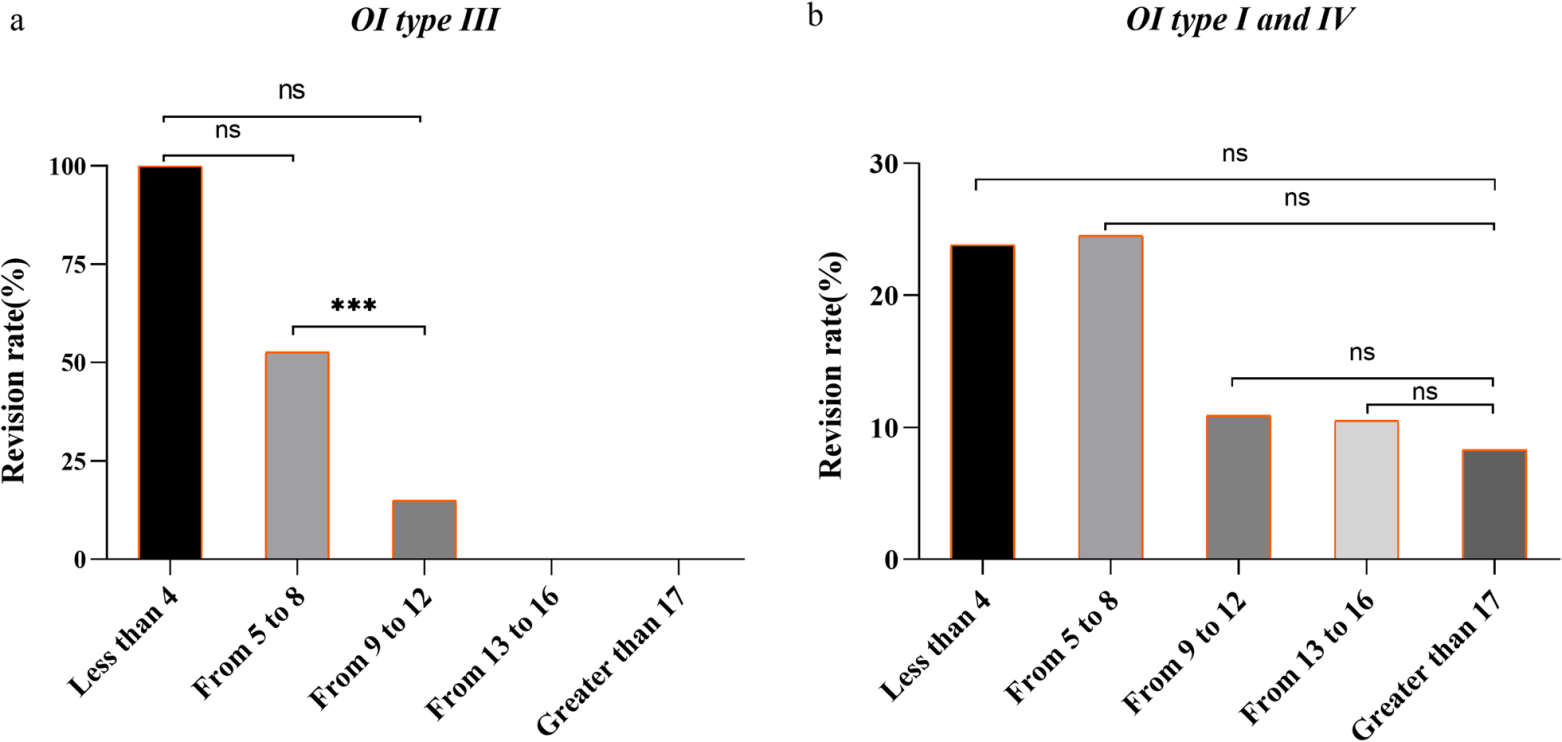
The revision rate of 211 patients with type III OI (**a**) and types I and IV OI (**b**) divided into 5 groups based on the initial age at surgery

All patients with type III OI who were aged less than or equal to 4 years underwent revision; 56.5% of patients with type III OI who were aged from 5 to 8 years underwent revision; 9.5% of patients who were aged from 9 to 12 years underwent revision; no patients aged from 13 to 16 years underwent revision, and no patients aged greater than or equal to 17 years underwent revision. There were significant differences in the impact at age of initial operation on postoperative revision in patients with type III OI (*P* < 0.001, Fig. 2a). There was no significant difference between patients aged less than or equal to 4 years old and patients aged from 5 to 8 years old (*P* = 0.50) (Fig. 2a). There was no significant difference between patients aged less than or equal to 4 years old and patients aged from 9 to 12 years old (*P* = 0.024) (Fig. 2a). There were significant differences between patients aged from 5 to 8 years old and patients aged from 9 to 12 years old (*P* = 0.001) (Fig. 2a). There was no significant difference in the impact at age of initial operation on postoperative revision between type I and type IV patients (Fig. 2b). The revision rate of surgery was the lowest for patients with type III OI who were aged 9 to 12 years old.

**Fig. 3:**
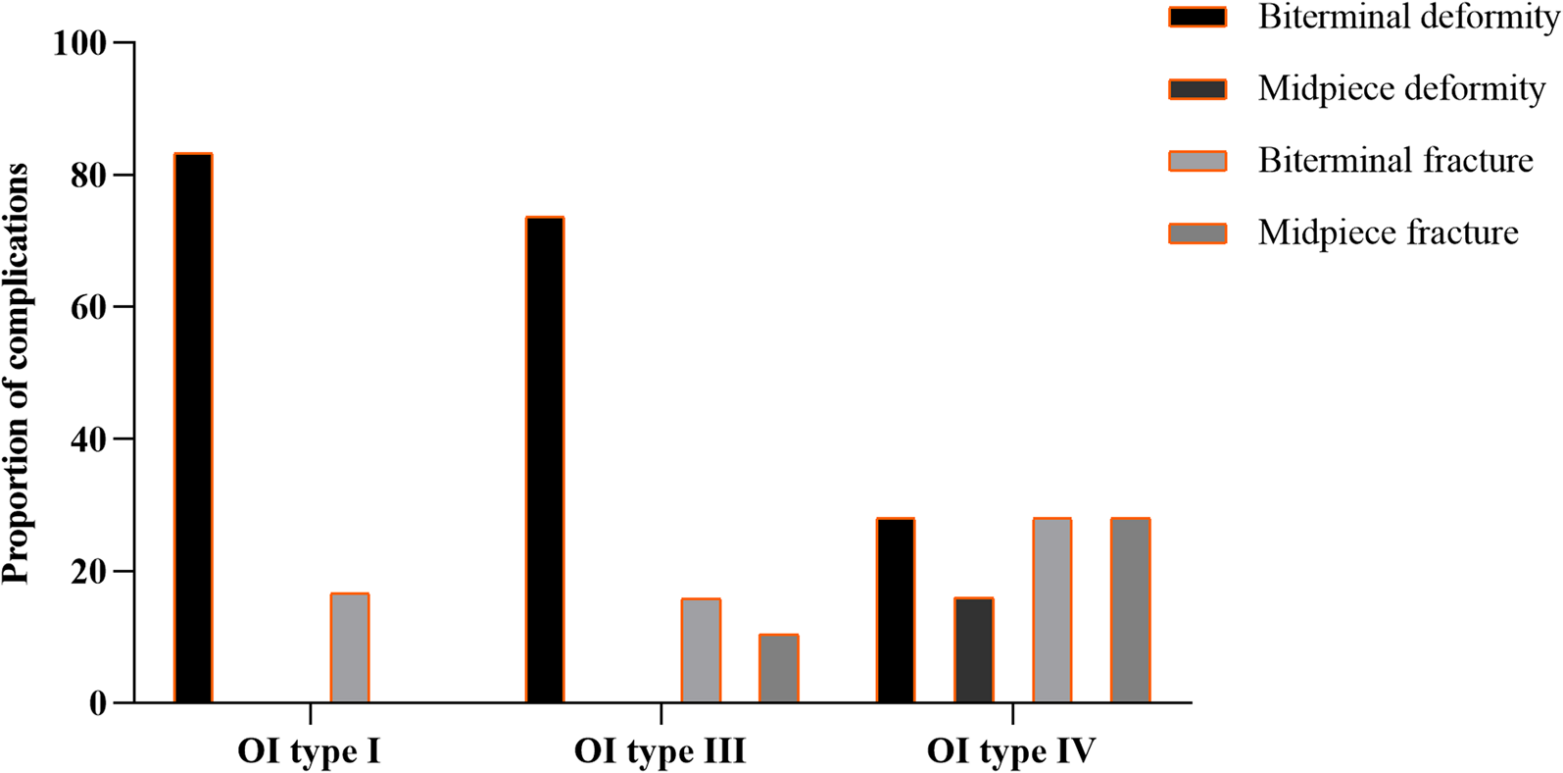
The proportion of complications included biterminal deformity, biterminal fracture, midpiece deformity, and midpiece fracture in patients with OI

**Fig. 4:**
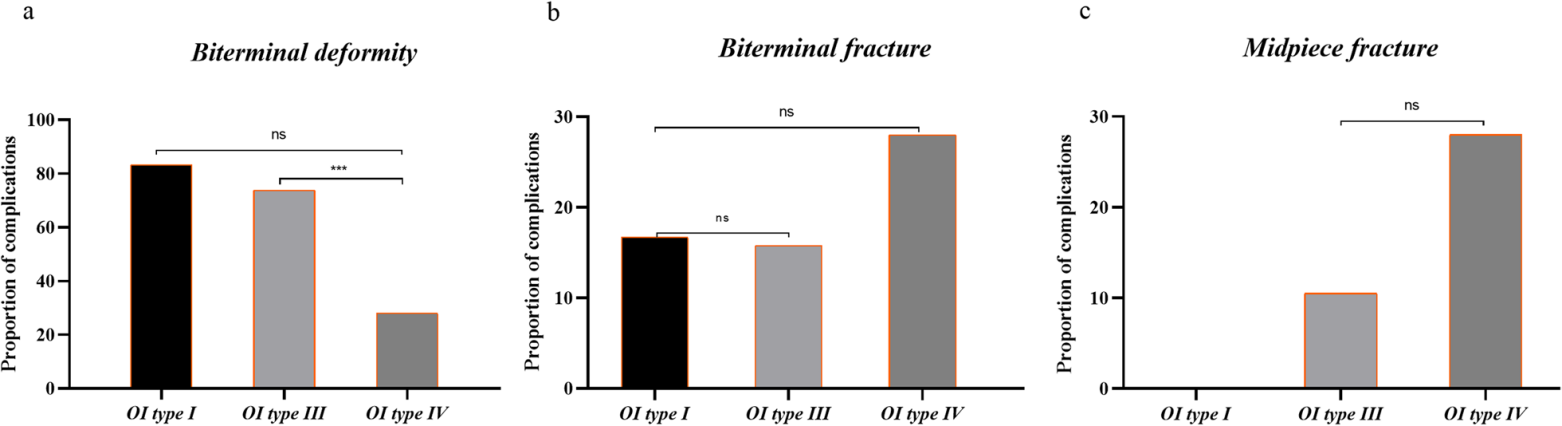
Detailed figure of proportions of the complications in patients with OI. **a** The proportions of biterminal deformity in patients with OI. **b** The proportions of biterminal fracture in patients with OI. **c** The proportions of midpiece fracture in patients with OI

Out of the 211 patients, we recorded 50 complications: 6 in type I, 19 in type III and 25 in type IV OI (Fig. 3). Biterminal deformity accounted for the vast majority of complications in patients with OI of type I and type III, and the four categories of complications were relatively uniform in patients with OI of type IV. Classification mainly affected the incidence of the deformity as one of the postoperative complications, and there was a significant difference in the rate of this complication between patients with type III and type IV OI (*P* = 0.003) (Fig. 4).

## Discussion

This study explored the patient-related factors affecting the revision procedures of tibia in patients with osteogenesis imperfecta treated with the Peter-Williams nail and investigated the relationship between the risk factors and complications. We found that the age at initial surgery and classification had a statistically significant influence on the revision rate, and the revision rate was lower for type III patients aged 9 to 12 years old. The study also reported a higher incidence of postoperative deformity in patients with type III OI.

The treatment objectives for OI include reducing the incidence of fractures, improving pain, and promoting growth, activity, and functional independence. However, it is important to note that the vast majority of OI patients are children whose bones are still in the growth stage. It usually needs to be revised again to complete functional reduction for the tibia of people with OI because of fracture or deformity occurring a few years after surgical treatment [12].

Compared with traditional children with fractures, fixation with plates and elastic nails was not suitable for children with OI. Enright WJ et al. discouraged the use of plates for long bones because of the increasing risk of fracture of the plate's edge [13]. Popkov et al. confirmed that elastic nails were ineffective and prone to revision when used in patients with OI [14]. Fractures were the most common complications occurring at the middle of the elastic nail where the stress was concentrated. Sterian A et al. recently reported fewer complications related to the anchorage system with the use of the Fassier-Duval rod in tibial fracture of patients with OI [15, 16]. However, the extension of the Fassier-Duval rod required an incision from the arthrotomies at the ankle and knee, which caused multiple injuries to the joint [17]. Moreover, the inner core thread of the Fassier-Duval rod cannot be fixed effectively by the distal tibial bone, especially for younger children with a small diameter of the medullary cavity [18]. The Peter-Williams nail had more unique advantages in tibial orthopaedic treatment than other implants, such as the Rush nail, Kirschner wire and telescopic rod. Insertion of the Peter-Williams nail does not require an incision from the arthrotomies at the knee, which causes few injuries to the joint. The intramedullary nail remained completely in the medullary cavity rather than left outside the cortex of bone compared with the Rush nail and Fassier-Duval rod. Moreover, the Peter-Williams nail was associated with relatively low cost and high convenience to for reoperation [19].

Above all, different implants had different risks of revision for patients with OI because of the various mechanisms of action and associated complications. When patients underwent stabilization with plates and screws, complications including screw loosening and fracture around the plate were reported [13]. When patients underwent operations with elastic and non-elongating rods, the incidence of complications was high, and the revision rate was high [14, 20]. Moreover, patient-related factors can also increase the risk of revision and complications in patients with OI. In our cohort, the risk of revision was found to be correlated with the classification of OI and initial operation age. However, interestingly, the preoperative tibial deformity angle cannot affect the revision rate, which seems inconsistent with the traditional concept [21]. It is speculated that the preoperative bending angle may not only be related to the classification but also be relevant to objective factors including the living environment. The data of 211 patients were divided into five different cohorts based on variations in the distribution of osteogenesis imperfecta types and characteristics of bone growth and development in children. During puberty (characterized by an age of 12 years old), the fracture rate is high, which plays a fundamental role in the accumulation of bone mass [22].

The current study found that classification was an influencing factor on revision procedures, which was consistent with the finding that patients with type III OI were more likely to have progressive deformity after the initial surgery. Type III OI patients had a greater degree of bone fragility and were more likely to develop progressive deformity after surgery, which usually required revision [23]. In our cohort, a higher risk of deformity was found in type III OI patients than in type I and type IV OI patients. Van Dijk FS et al. reported that individuals with type III OI usually have newborn or infant presentation with bone fragility and multiple fractures leading to progressive deformity of the skeleton [24], which was consistent with the data collected in our study.

The age of initial surgery was also an influencing factor on revision procedures, because bone mineral density and bone fragility changed at different ages. There was no consensus on the ideal age for surgery. The incidence of complications in intramedullary fixation was higher among those under 5 years of age, than among older children. In addition, bone growth was the fastest during the first years of life, and the need for further surgery (revision) was obvious [1, 25].

Additional limitations of the obvious study stem from its duration of follow-up (which was only short to medium-term in some studies), which could have introduced recall bias and the small number of patients. This study breaks through the limitation with a long duration of follow-up of 8–10 years and big data of patients even OI is a rare disease, especially in tibia. Our study can provide additional clinical data for tibial surgery in patients with osteogenesis imperfecta and evidence that the age of initial surgery and classification affect the revision rate of tibial surgery with the Peter-Williams nail.

## Conclusion

The study reported that the age of initial surgery and classification were the influencing factors in revision procedures on tibia of patients with osteogenesis imperfecta treated with the Peter-Williams nail and appropriate surgical treatment at the age of 9–12 years old can reduce the revision rate in patients with type III OI. The current study also reported a higher incidence of deformity in patients with type III OI.

## Supplementary Information

**Additional file 1.** The preoperative radiograph and postoperative radiograph of the Peter-Williams nail.

## Data Availability

The datasets used and analysed during the current study are available from the corresponding author on reasonable request.

## Abbreviations

OI: Osteogenesis imperfecta
IQR: Interquartile range
CI: Confidence interval

## References

[1] Sinikumpu JJ, Ojaniemi M, Lehenkari P, Serlo W (2015). Severe osteogenesis imperfecta Type-III and its challenging treatment in newborn and preschool children. A systematic review. Injury.

[2] Sillence DO, Rimoin DL, Danks DM (1979). Clinical variability in osteogenesis imperfecta-variable expressivity or genetic heterogeneity. Birth Defects Orig Artic Ser.

[3] Forlino A, Marini JC (2016). Osteogenesis imperfecta. The Lancet.

[4] Marini JC, Forlino A, Cabral WA, Barnes AM, San Antonio JD, Milgrom S (2007). Consortium for osteogenesis imperfecta mutations in the helical domain of type I collagen: regions rich in lethal mutations align with collagen binding sites for integrins and proteoglycans. Hum Mutat.

[5] Ben Amor IM, Roughley P, Glorieux FH, Rauch F (2013). Skeletal clinical characteristics of osteogenesis imperfecta caused by haploinsufficiency mutations in COL1A1. J Bone Miner Res.

[6] Georgescu I, Vlad C, Gavriliu T, Dan S, Pârvan AA (2013). Surgical treatment in osteogenesis imperfecta—10 years experience. J Med Life.

[7] Trejo P, Rauch F (2016). Osteogenesis imperfecta in children and adolescents-new developments in diagnosis and treatment. Osteoporos Int.

[8] Nicolaou N, Bowe JD, Wilkinson JM, Fernandes JA, Bell MJ (2011). Use of the Sheffield telescopic intramedullary rod system for the management of osteogenesis imperfecta: clinical outcomes at an average follow-up of nineteen years. J Bone Joint Surg Am.

[9] Yang H, Li B, Xing C, Gao S, Zhu W, Xiong Y (2023). Which is the best femoral implant in children with osteogenesis imperfecta? a retrospective cohort study of 783 procedures. BMC Musculoskelet Disord.

[10] Jerosch J, Mazzotti I, Tomasevic M (1998). Complications after treatment of patients with osteogenesis imperfecta with a Bailey-Dubow rod. Arch Orthop Trauma Surg.

[11] Zionts LE, Ebramzadeh E, Stott NS (1998). Complications in the use of the Bailey-Dubow extensible nail. Clin Orthop Relat Res.

[12] Middleton RW (1984). Closed intramedullary rodding for osteogenesis imperfecta. J Bone Joint Surg Br.

[13] Enright WJ, Noonan KJ (2006). Bone plating in patients with type III osteogenesis imperfecta: results and complications. Iowa Orthop J.

[14] Popkov D, Popkov A, Mingazov E (2019). Use of sliding transphyseal flexible intramedullary nailing in pediatric osteogenesis imperfecta patients. Acta Orthop Belg.

[15] Sterian A, Balanescu R, Barbilian A, Tevanov I, Carp M, Nahoi C (2015). Early telescopic rod osteosynthesis for osteogenesis imperfecta patients. J Med Life.

[16] Sterian A, Balanescu R, Barbilian A, Ulici A (2015). Osteosynthesis in Osteogenesis Imperfecta, telescopic versus non-telescopic nailing. J Med Life.

[17] McClure PK, Franzone JM, Herzenberg JE (2022). Challenges with Fassier-Duval rod exchanges in congenital pseudarthrosis of the tibia: explant roadblock and solution. J Pediatr Orthop B.

[18] Birke O, Davies N, Latimer M, Little DG, Bellemore M (2011). Experience with the Fassier-Duval telescopic rod: first 24 consecutive cases with a minimum of 1-year follow-up. J Pediatr Orthop.

[19] Imajima Y, Kitano M, Ueda T (2015). Intramedullary fixation using Kirschner wires in children with osteogenesis imperfecta. J Pediatr Orthop.

[20] Gamble JG, Strudwick WJ, Rinsky LA, Bleck EE (1988). Complications of intramedullary rods in osteogenesis imperfecta: Bailey-Dubow rods versus nonelongating rods. J Pediatr Orthop.

[21] Javaid MK, Mordenti M, Boarini M, Sangiorgi L, Group EBW, Westerheim I (2021). Patients' priorities and expectations on an EU registry for rare bone and mineral conditions. Orphanet J Rare Dis.

[22] Saggese G, Baroncelli GI, Bertelloni S (2002). Puberty and bone development. Best Pract Res Clin Endocrinol Metab.

[23] Persiani P, Ranaldi FM, Martini L, Zambrano A, Celli M, D'Eufemia P (2019). Treatment of tibial deformities with the Fassier-Duval telescopic nail and minimally invasive percutaneous osteotomies in patients with osteogenesis imperfecta type III. J Pediatr Orthop B.

[24] Van Dijk FS, Sillence DO (2014). Osteogenesis imperfecta: clinical diagnosis, nomenclature and severity assessment. Am J Med Genet A.

[25] Oznur A, Tokgozoglu AM, Alpaslan AM (1999). Complications in the use of the Bailey–Dubow extensible nail. Clin Orthop Relat Res.

