# Supplementary material for: The patient-related factors in revision procedures on tibia of patients with osteogenesis imperfecta treated with the Peter-Williams nail
Source: J Orthop Surg Res. 2023 Jul 26;18:532. doi: 10.1186/s13018-023-03952-w (PMC10373316; doi:10.1186/s13018-023-03952-w)
Supplement: Supplementary file 1 — Additional file 1. The preoperative radiograph and postoperative radiograph of the Peter-Williams nail. [file 13018_2023_3952_MOESM1_ESM.docx]

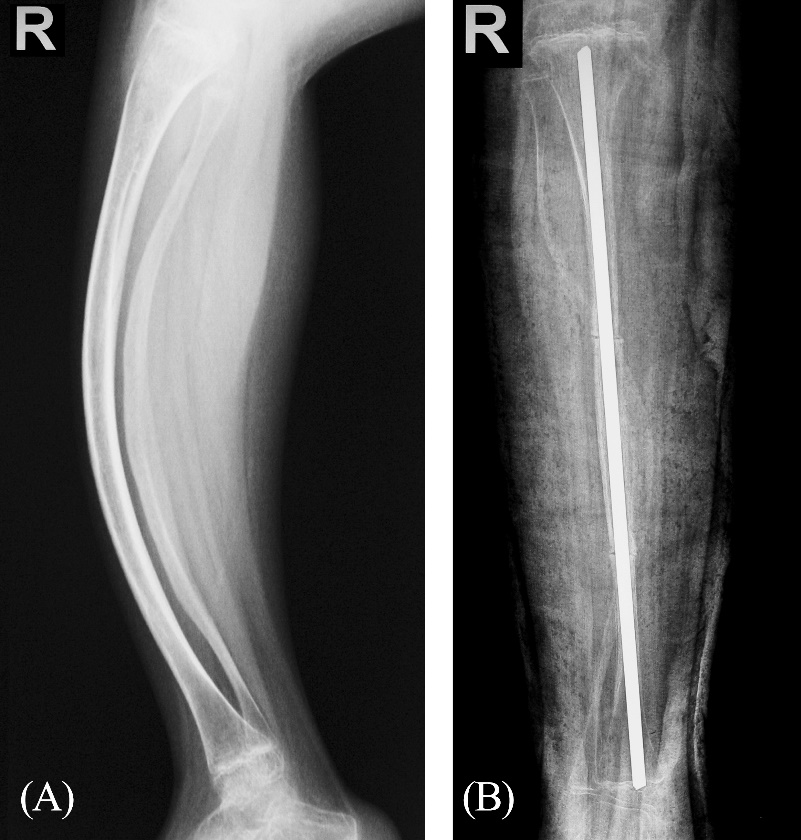


**Supplementary figure 1**

**Supplementary Fig. 1** The preoperative radiograph (A) and postoperative radiograph

(B) of the Peter-Williams nail. Deformity happened in the middle of the tibia and was

corrected completely with Peter-Williams nail.
